# IKT Guiding Principles: demonstration of diffusion and dissemination in partnership

**DOI:** 10.1186/s40900-023-00462-1

**Published:** 2023-07-12

**Authors:** Alanna Shwed, Femke Hoekstra, DivyaKanwar Bhati, Peter Athanasopoulos, John Chernesky, Kathleen Martin Ginis, Christopher B. McBride, W. Ben Mortenson, Kathryn M. Sibley, Shane N. Sweet, SCI Guiding Principles Panel, Heather L. Gainforth

**Affiliations:** 1 School of Health and Exercise Sciences, University of British Columbia, Kelowna, BC Canada; 2 International Collaboration on Repair Discoveries (ICORD), University of British Columbia, Vancouver, BC Canada; 3 Spinal Cord Injury Ontario, Toronto, ON Canada; 4 Praxis Spinal Cord Institute, Vancouver, BC Canada; 5 Spinal Cord Injury British Columbia, Vancouver, BC Canada; 6 Department of Occupational Science and Occupational Therapy, Faculty of Medicine, University of British Columbia, Vancouver, BC Canada; 7 Rehabilitation Research Program, Vancouver Coastal Health Research Institute, Vancouver, BC Canada; 8 Rady Faculty of Health Sciences, University of Manitoba, Winnipeg, MB Canada; 9 Department of Kinesiology and Physical Education, McGill University, Montreal, QC Canada

**Keywords:** Knowledge mobilization, Research co-creation, Integrated knowledge translation, Research partnership, Implementation science, Dissemination

## Abstract

**Introduction:**

Integrated knowledge translation (IKT) is a partnered approach to research that aims to ensure research findings are applied in practice and policy. IKT can be used during diffusion and dissemination of research findings. However, there is a lack of understanding how an IKT approach can support the diffusion and dissemination of research findings. In this study, we documented and described the processes and outcomes of an IKT approach to diffusing and disseminating the findings of consensus recommendations for conducting spinal cord injury research.

**Methods:**

Communication of the IKT Guiding Principles in two phases: a diffusion phase during the first 102 days from the manuscript’s publication, followed by a 1147 day active dissemination phase. A record of all inputs was kept and all activities were tracked by monitoring partnership communication, a partnership tracking survey, a project curriculum vitae, and team emails. Awareness outcomes were tracked through Google Analytics and a citation-forward search. Awareness includes the website accesses, the number of downloads, and the number of citations in the 29 month period following publication.

**Results:**

In the diffusion period, the recommendations were viewed 60 times from 4 different countries, and 4 new downloads. In the dissemination period, the recommendations were viewed 1109 times from 39 different countries, 386 new downloads, and 54 citations. Overall, during dissemination there was a 17.5% increase in new visitors to the website a month and a 95.5% increase in downloads compared to diffusion.

**Conclusion:**

This project provides an overview of an IKT approach to diffusion and dissemination. Overall, IKT may be helpful for increasing awareness of research findings faster; however, more research is needed to understand best practices and the the impact of an IKT approach on the diffusion and dissemination versus a non-partnered approach.

**Supplementary Information:**

The online version contains supplementary material available at 10.1186/s40900-023-00462-1.

## Abstract (plain language summary)

Often, research findings do not get to the people, groups, and/or institutions who could benefit from the findings. Two ways to help move research into practice more efficiently are to: (1) work in partnership with people who may benefit most from the research findings, and (2) share and communicate the findings of research beyond scientists. However, little is known about how to work in partnership while sharing the results of a research study. Therefore, this project demonstrates how a research partnership can work while promoting the results of their research project. Overall, working in partnership while sharing research findings may further help to ensure the research results are shared with those who could benefit from those findings.

**Supplementary Information:**

The online version contains supplementary material available at 10.1186/s40900-023-00462-1.

## Introduction

Delays or failures in applying research findings in practice or policies have been reported in research domains such as education [1], psychology [2], and health [3–5]. As a result, greater focus has been directed towards approaches and activities that aim to help move research findings into practice [6]. For example, diffusion and dissemination are the stages of research that refer to the communication and activities dedicated to sharing of research findings. While similar, diffusion refers to the natural spread of research findings and dissemination refers to conscious efforts to spread new knowledge, policies, and practices to target audiences [7].

Research partnerships are a promising avenue for helping move research findings into practice [8, 9]. Integrated knowledge translation (IKT) is one research partnership approach that requires the two-way sharing of knowledge that is motivated by the application of research findings in practice [9]. IKT has been defined as the meaningful engagement of the right knowledge user at the right time throughout the research process [10, 11]. Both researchers and knowledge users are experts and there is an expectation of shared decision-making [12, 13]. IKT is an approach to partnership motivated by the application of research findings in practice.

While IKT is a broadly accepted approach for addressing research-practice gaps, the science and practice of IKT are still in their infancy [11, 14]. There remains a need to understand if, why, and how IKT has impact on moving research findings into practice. Research agendas include calls for studies that report on the processes (e.g., inputs, activities, outputs) and outcomes of an IKT approach to research [14, 15]. Further, there are calls for the integration of partnered research with diffusion and dissemination practices to allow for the communication and eventual application of research *with* key knowledge users instead of *on* key knowledge users [16].

While research partnerships span diverse research domains, identities, locations, and/or contexts with a strong focus on research that serves equity-deserving group, concerns about tokenism in research partnerships have been raised [13, 17, 18]. Tokenism is when a knowledge user is asked to endorse, and therefore legitimize, a research program over which they will have little-to-no decision-making power [13, 17, 18]. To combat tokenism and to begin address gaps in the science and practice of IKT, a multidisciplinary partnership of researchers, research users, and funders co-developed consensus recommendations for conducting and disseminating spinal cord injury (SCI) research in partnership. We refer to these as the IKT Guiding Principles (see www.IKTprinciples.com). This partnership used an IKT approach and systematic and rigorous methods to synthesize research findings from a review of reviews [19], a scoping review [20], and interviews with SCI researchers and knowledge users who have experience in research partnerships [21]. The resulting eight principles outline norms, rules, or beliefs for research partnerships to consider when conducting SCI research in partnership and aim to directly address concerns about tokenism in the SCI research system [13].

To support awareness and uptake of the principles as well as meaningful research partnerships within the SCI research system, the IKT Guiding Principles partnership co-developed a strategic diffusion and dissemination plan. Diffusion and dissemination of the principles provides an ideal opportunity to advance the science and practice of IKT by reporting and comparing differences of an IKT approach to diffusion and dissemination processes and outcomes. Therefore, this descriptive evaluation study aimed to: [1] describe the processes of an IKT approach to diffusion and dissemination of the IKT Guiding Principles; and [2] identify differences in awareness outcomes between a principled partnered approach to diffusion and a principled partnered approach to dissemination. It was hypothesized that a principled partnership approach to dissemination effort as compared to diffusion efforts would yield greater and faster awareness of the IKT Guiding Principles.

## Methods

### Resarch design

This project and the partnership’s overarching paradigm is pragmatism [22, 23]. Pragmatism has the primary aim of using tangible findings to solve practical ‘real-world’ problems [22, 23]. A logic model was developed to describe diffusion and dissemination inputs, activities, outputs, and outcomes.

### Partnership paradigm and context

At the time of diffusion and dissemination, the IKT Guiding Principles Partnership reported on in this paper included 22 members who are researchers and/or knowledge users. The partnership consists of members affiliated with 16 different community and/or research organizations/universities, who all have multiple roles in the SCI research system (see Additional file 1).

The partnership established a governance structure to clarify members’ roles and decision-making processes (see Additional file 2). The governance structure was co-developed to ensure two-way communication, intellectual leadership, and collaboration between all members of the partnership. A team was established to support the strategic diffusion and dissemination of the recommendations. Members of the team were strategically selected to include leaders with experience in SCI research, knowledge translation, graphic design, community advocacy, and public policy.

### Diffusion and dissemination plan

To design the diffusion and dissemination plan, our partnership used Lavis’ and colleagues’ organizing framework [24] as Barwick and colleagues’ Knowledge Translation Planning Template [25]. Table 1 summarizes the diffusion and dissemination plan. We used these frameworks to consider and identify target audiences, main messages, messengers, goals, and strategies (see Additional file 3). Using an IKT approach to diffusion and dissemination required our partnership to define our partners’ level of commitment and engagement in the diffusion and dissemination phases. The partnership accommodated all levels of partner engagement to avoid overburdening partners, especially those whose main job is not research or this project. For example, some partners wanted to only provide their higher-level feedback on dissemination activities, whereas others wanted to be part of working meetings to co-create the tools and resources alongside the PI and the graduate trainees.

**Table 1:** Diffusion and dissemination planning template of the IKT Guiding Principles

	Diffusion	Dissemination
Identified knowledge users	SCI Researchers	SCI Researchers SCI Knowledge users (e.g., persons with lived experience, policymakers, health and/or service providers, professional organizations, industry partners, etc.) SCI Research funders
Main messages	The eight Integrated Knowledge Translation (IKT) Guiding Principles were developed to support partnerships to conduct quality and ethical research in spinal cord injury (SCI) that is relevant, useful, useable, and avoids tokenism. These principles can be considered and/or used by all partners (researchers, knowledge users, and funders of SCI research) early and throughout the entire research process. Partners should regularly refer to the IKT Guiding Principles while reflecting on their approach, contributions, and commitment to the partnership and adjust as needed.	
KT goals	Generate awareness	
KT strategies	To generate awareness: Publication Social media	To generate awareness: Materials (toolkit, pamphlet) Plain language summaries Workshop, webinar Conferences Social Media Media Network
KT process	IKT approach to end of grant KT.	
KT evaluation	Awareness indicators: 1. IKT Guiding Principles website visits 2. Downloads of the IKT Guiding Principles 3. Citations of the IKT Guiding Principles	
Resources	Human resource: Open Access Publication Web Support: Social media	Governing Board: Governance structure; KT Team Financial Support: SSHRC funding; Michael Smith funding In-kind resources from partners and University Human Support: Partner networks; Graduate trainees research assistance; Graphic design; Institution media Web Support: Webinars; Website; Social media

Our partnership was particularly interested in understanding the added value of comparing the partnership engagement and awareness outcomes during diffusion and dissemination. Therefore, the partnership chose to undertake communication of the IKT Guiding Principles in two phases. The first phase was diffusion which occurred from October 28th, 2020, to February 7th, 2021. These activities included a journal manuscript publication and a few Twitter postings. Dissemination activities occurred from February 8th, 2021, and are ongoing (this paper reports until March 31st, 2023). These activities have included project branding, many social media posts (Twitter and Facebook), webinars, project website, media releases, invited talks, information videos, and conference and community presentations.

### Process measures

#### Inputs

Inputs are defined as the human, financial, organizational, community, etc. resources that need to be invested so that the program (i.e., diffusion and dissemination of the IKT Guiding Principles) can be performed [26]. Inputs were tracked by the first author (AS) from the start of diffusion through to the present of dissemination. A record of all inputs was kept and updated when a new end of grant activity took place. Table 2 provides an overview of the inputs from a partnered approach to diffusion and dissemination of the IKT Guiding Principles.

**Table 2:** Inputs needed for diffusion and dissemination of the IKT Guiding Principles

	Diffusion	Dissemination
Financial	SSHRC funding Michael Smith Funding	SSHRC funding Michael Smith Funding
Human	IKT Guiding Principles partnership time	KMb Team expertise IKT Guiding Principles partnership time and network channels Graduate student research assistant time Graphics and website designers UBC media people
Technology	Google Analytics to track awareness outcomes	Qualtrics, Google Analytics, Zoom, UBC CMS Website platform
Physical Space	N/A	Room space for in-person meetings
Social Media	Twitter	Twitter and Facebook

#### Activities

Activities are defined as what the program (i.e., diffusion and dissemination of the IKT Guiding Principles) does with the inputs [26]. Four data sources were used to measure activities: [1] Partnership communication, [2] Partnership tracking survey, [3] Project CV, and [4] Partner emails.

##### Partnership communication

Monitoring of all meetings, emails, and decisions were tracked by the first author (AS) through Zoom partnership meeting recordings, meeting minutes, saved emails, and calendar invites.

##### Partnership tracking survey

A tracking survey was sent out four times to the entire IKT Guiding Principles partnership by the partnership lead (HG) and yielded 12 responses from 9 different partners. The survey asked for details about all diffusion and dissemination activities related to the IKT Guiding Principles such as type, dates, numbers (e.g., emails, papers), target audiences (e.g., people, organizations). Refer to Open Science Framework for the full survey.

##### Project CV

Findings collected from all four tracking surveys were combined to create the CV to help partners identify any missing activities. The project CV was sent out to all partners to elicit additional information and/or corrections to the presented dissemination activities.

##### Partner emails

The partnership lead (HG) sent out formal updates about diffusion and dissemination activities (e.g., presentations, publications), achievements (e.g., awareness numbers), and challenges (e.g., tracking response rates) to elicit feedback on any missing information.

### Outcome measures

The primary outcome of interest was awareness of the IKT Guiding Principles. Awareness was defined as the number of views of the IKT Guiding Principles. Awareness was measured using [1] Google Analytics, and [2] Citation forward search. First, Google Analytics of the IKT Guiding Principles website (www.IKTprinciples.com) tracked the number of new people that accessed the website and the number of new people that downloaded the principles. Views were excluded if they did not stay on the website for 10 s or longer or if they had visited the website before (i.e., not a new visitor). Second, a citation forward search (i.e., identifying articles that cite original work after it has been published) was conducted with Google Scholar and through the Archives of Physical Medicine and Rehabilitation. All peer-reviewed manuscripts and grey literature that cited the IKT Guiding Principles in any capacity, after their initial publication on October 28th, 2020 through March 31st, 2023, were identified. Both data sources were consolidated to create an overall awareness figure that is presented in the findings. The rate of awareness was calculated by averaging the number of new visitors to the IKT Guiding Principles website, new downloads, and new citations of the principles each month. We then compared those averages to determine the percentage increase of awareness during dissemination compared to diffusion (project aim 2). We also calculated and compared the average rate of awareness (website visits, downloads, and citations) during diffusion (November 2020–February 2021), first year of dissemination (February 2021–March 2022), and second year of dissemination (February 2022–March 2023).

## Results

### Outputs

**Fig. 1:**
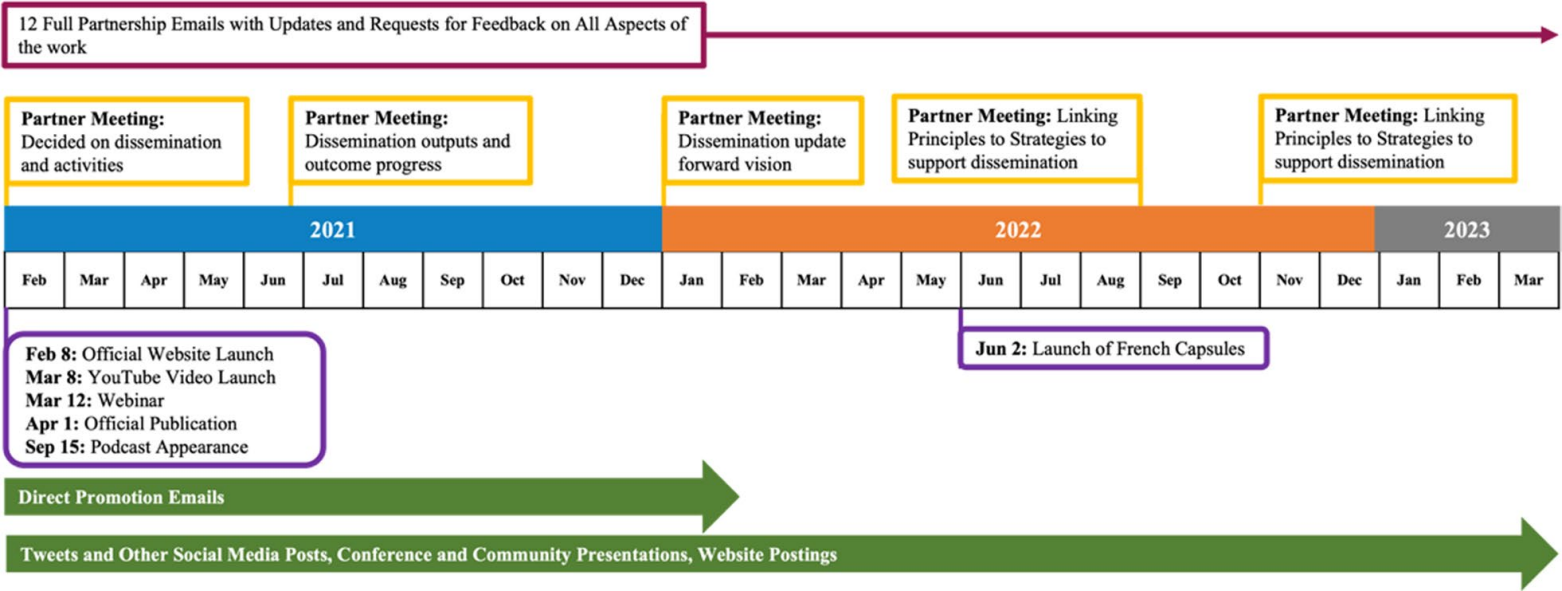
Outputs from disseminating the IKT Guiding Principles

Figure 1 pressents the difference in outputs from a IKT approach to diffusion and a IKT approach to dissemination of the IKT Guiding Principles. All four data sources that tracked activities were consolidated to create a timeline understanding of the diffusion and dissemination outputs.

#### Diffusion

Diffusion took place from October 2020 to February 2021 to ensure initial dissemination tools were prepared adequately (i.e., IKT Guiding Principles pamphlet, informational video, etc.). Only the official manuscript of the principles was available online. From September 2020 to January 2021, the partnership met virtually twice to discuss the diffusion of the IKT Guiding Principles and there were three partnership emails sent out by the partnership lead (HG) that communicated project updates and plans for next steps. Diffusion of the principles included a tweet about the publication by the journal and some members of the partnership; however, no other activities were conducted.

#### Dissemination

From February 2021 to March 2023, the partnership met in-person and virtually, and consistently communicated over email to plan dissemination activities (e.g., branding, activities, timeline). During that time, there were 5 partnership meetings specific to dissemination to make decisions about next steps, and 12 partnership emails that communicated project updates and plans for next steps. These partnership meetings and emails do not include the many smaller email chains and meetings that happened throughout the project with main partners (KT team) involved.

Once the main tools for dissemination (i.e., website, branding, and bilingual Principles 1-page summary) were ready, dissemination of the principles began. A formal launch (i.e., a planned week with specific promotion activities each day) took place February 8–12th, 2021, and involved coordinated efforts from the IKT Guiding Principles partnership (e.g., webinar, personal emails). Dissemination activities have continued since; however, less frequent after that first week.

### Awareness outcomes

Below we present the difference in awareness outcomes between a principled partnered approach to diffusion and dissemination of the IKT Guiding Principles.

#### Diffusion

During diffusion (November 2020 through January 2021), the IKT Guiding Principles were accessed by 60 new people (i.e., new users of the IKT Guiding Principles website) in 4 different countries, downloaded 4 times, and cited once. Overall, diffusion of the IKT Guiding Principles resulted in ~ 20 new visitors/month, ~ 1 downloads a month, and ~ 0.3 citations a month. The average rate of awareness during diffusion was 1.71% for website visits, 0.34% for downloads, and 0.62% for citations.

#### Dissemination

**Fig. 2:**
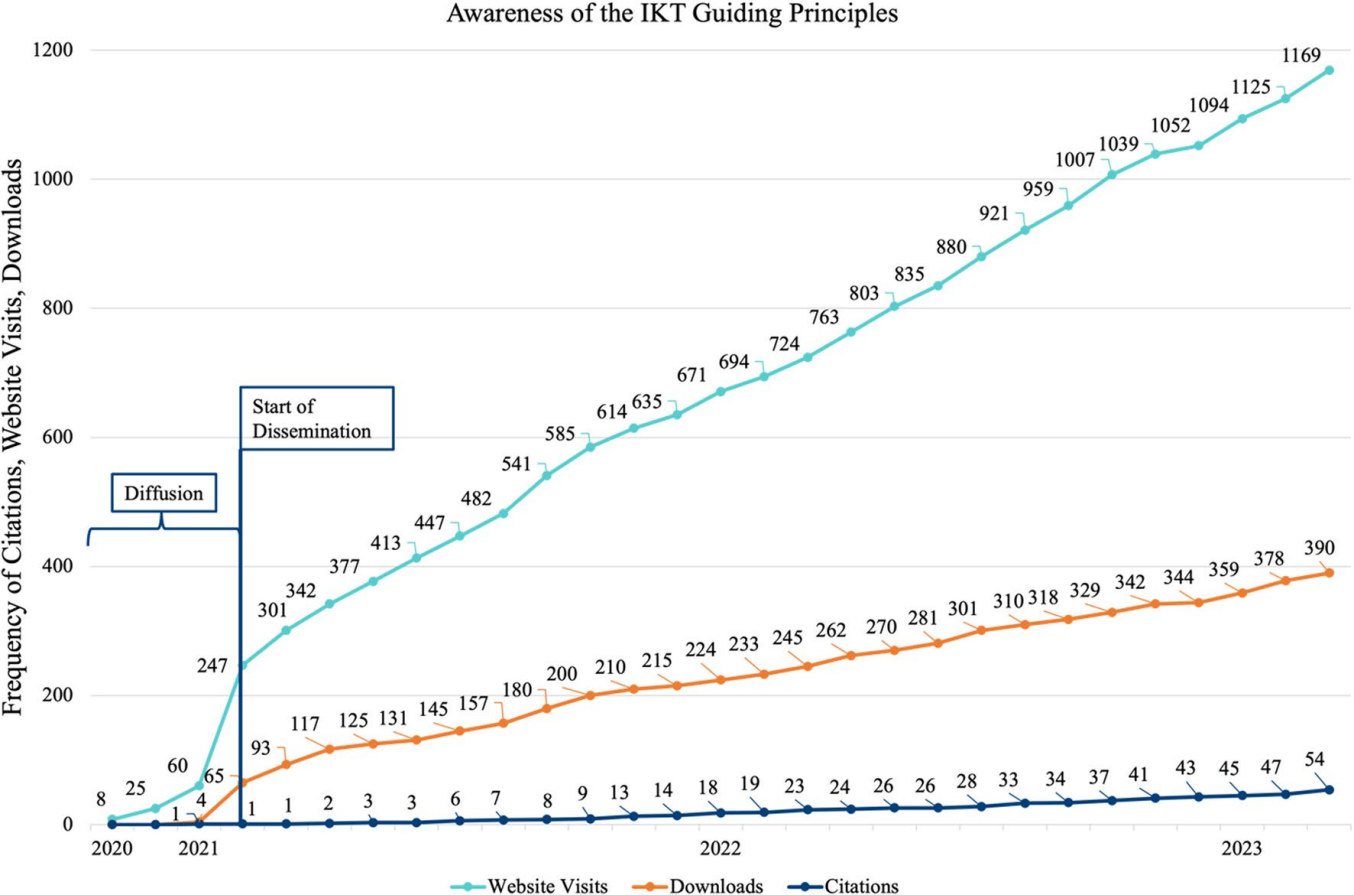
Awareness of the IKT Guiding Principles

During dissemination from February 2021 through March 2023, the IKT Guiding Principles were accessed by 1109 new people (i.e., new users of the IKT Guiding Principles website) in 39 different countries, downloaded 386 times, and cited 54 times (35 citations from IKT Guiding Principles partnership team). Overall, a partnered approach to dissemination of the IKT Guiding Principles resulted in ~ 42 new visitors a month, ~ 9 downloads a month, and ~ 2 citations a month. There was a 17.5% increase in new visitors to the website a month and a 95.5% increase in downloads a month from diffusion to dissemiantion. Figure 2 demonstrates the frequency increase in awareness of the IKT Guiding Principles during diffusion and dissemination.

The average rate of awareness during dissemination averaged 3.65% for website visits, 3.81% for downloads, and 3.77% for citations. This demonstrated a 1.94% increase in rate of awareness from website visits, 3.46% increase in rate of awareness from downloads, and 3.16% increase in rate of awareness from citations during dissemination compared to diffusion.

## Discussion

This project demonstrated the processes (i.e., inputs, activities, outputs) and outcomes (i.e., awareness) of a principled partnered approach to diffusion and dissemination of the IKT Guiding Principles. When a partnered approach to dissemination was used the rate of awareness of the IKT Guiding Principles was higher than during the diffusion phase. Findings from this work will inform future work related to disseminating the IKT Guiding Principles and provide a roadmap for other partnerships who are working in partnership during end-of-grant KT.

The largest increase in awareness of the IKT Guiding Principles happened during the formal launch week which involved planned and unique dissemination activities every day of the week (e.g., Tweets, emails, website launch). These findings support the call for research partnerships to do more beyond solely traditional diffusion efforts (e.g., publishing scientific research findings) when sharing their work [27]. Researchers and research partnerships are encouraged to use diverse strategies for dissemination (i.e., promoting work that is digestible and accessible to wider audiences than just scholars) [27].

While it is important to acknowledge the call from the research system for improved diffusion and dissemination work from researchers [28, 29], it is also important to be cognisant of what is realistic. As demonstrated in this project, using a different dissemination activities often may be helpful for disseminating findings to more people at a faster rate [30]. However, this approach may not be feasible long term as an everyday approach. Many researchers, knowledge users, and funders have other job responsibilities or priorities and cannot maintain this level of dissemination effort. Researchers also may not have the skill required for effective dissemination as they are not trained in communications or journalism (i.e., sharing information about their research to an audience outside of their specialized field). Further, research teams will need to consider what research findings require a high level of dissemination effort and which ones do not. For example, not all single studies may need week-long dissemination plans, but larger synthesis of work may warrant that effort [31]. Given the success demonstrated in this project, research partnerships that determine high levels of dissemination efforts as important may consider planning to have more than one week (either together or spread out) of a dissemination activity each day. There is also a call for capacity-building within the research system to help foster consistent dissemination of research findings [32, 33]; capacity may include time, money, and designated dissemination roles.

### Suggestions for future research

While this work provides important information for advancing the science and practice of partnered research, more work is needed to understand how to best engage in and evaluate an IKT, or partnered research, approach. Overall, we do not have data to determine if an IKT approach to diffusion and dissemination is more effective than an unpartnered approach. Studies that are designed to compare an IKT approach to an unpartnered approach to diffusion and dissemination are also needed to understand the effectiveness of IKT. Further, whether an IKT approach, or different research partnership approach [16], to diffusion and dissemination leads to actual adoption, implementation, and transformation of the SCI research system is yet to be known. Longitudinal multiple case studies about how groups are using the principles or other partnership approaches, besides IKT, will be needed to answer these questions.

Further, while working in partnership is difficult [32], studying a partnership is also challenging and requires more research [11]. Work is needed to best understand how to monitor partnership processes and evaluate outcomes. Eliciting responses from the IKT Guiding Principles partnership, despite its members being motivated to do this work, was difficult as demonstrated by the few responses to the survey. This experience emphasizes the need for research to understand how to feasibly and accurately study partnerships while avoiding burden on partners.

Academic and research systems also need to recognize the importance of continued collaboration after the scientific paper is published (i.e., dissemination) for research findings to be impactful. With more support from the research system (e.g., dedicated funding for dissemination and partnership), research partnerships may be more inclined to continue efforts beyond research discovery through to dissemination and implementation. The research system needs to recognise the importance of dissemination activities and the resources that are required by researchers to share their work beyond a research publications (e.g., funding, training, qualified personnel). Finally, this paper discusses initial awareness of the IKT Guiding Principles. Future work looking at use and usefulness indicators as well as the partnership, policy, knowledge, attitude, and system change indicators [25] is needed to understand the impact of the principles in the SCI research system and beyond.

### Contribution and limitations

A major contribution of this paper is the example demonstrated of how to do IKT during the diffusion and dissemination stages of a research project. There are also limitations of this work that are acknowledged below. The first limitation are the gaps in the awareness outcomes. Google Analytics is an imperfect platform for identifying unique individuals as you cannot distinguish a person from an IP address. If the same person accessed the IKT Guiding Principles website or PDF document from two different IP addresses, then that person was counted as two different individuals. Conversely, if multiple people used the same computer (e.g., library, lab, etc.), they have all been considered the same person. Therefore, the awareness numbers are not exact as overestimation and underestimation are likely. However, we only counted new users in our metrics to help minimize overestimation as much as possible. Further, we do not have a full understanding of who our dissemination activities have reached. Future work evaluating our dissemination outcomes need to better understand who we are reaching to determine if more targeted approaches to specific groups (researchers, knowledge users, funders) is warranted.

Second, our citation forward search did not investigate the use of the IKT Guiding Principles. Citations of the principles tells us that people are aware of them (i.e., the goal of this paper); however, next steps are to understand their use which can be in part done by investigating how they are reported on in the literature. Third, there were limited responses to the partnership tracking survey and Project CV from members of the IKT Guiding Principles partnership. Limited responses mean we may not have captured all diffusion and dissemination outputs and may also suggest some partners may not have engaged in dissemination activities at all. There is a need for work that builds capacity for researchers, knowledge users, and funders to prioritize the work involved in sharing their research results. Building capacity requires institutional level practices, structures, cultures, and processes that enable, value, resource, support, and/or incentivize partnered research at every stage of the research process [32, 33].

## Conclusion

An IKT approach to diffusion and dissemination may be helpful for ensuring research findings are shared with those who may benefit from research findings. This project provides possible metrics and methodology for reporting on inputs, activities, outputs, and outcomes of an IKT approach to diffusion and/or dissemination. More work is needed to understand how to work in partnership during diffusion and dissemination to evaluate the effectiveness of a partnered approach.

## Supplementary Information

**Additional file 1.** IKT Guiding Principles Partnership and Expertise

**Additional file 2.** IKT Guiding Principles Partnership Governance Structure

**Additional file 3.** IKT Guiding Principles and Enacted Strategies

## Data Availability

The datasets supporting the conclusions of this article are available on OSF: https://osf.io/xc7j9.
