# Supplementary material for: IKT Guiding Principles: demonstration of diffusion and dissemination in partnership
Source: Res Involv Engagem. 2023 Jul 12;9:53. doi: 10.1186/s40900-023-00462-1 (PMC10337125; doi:10.1186/s40900-023-00462-1)
Supplement: Supplementary file 1 — Additional file 1. IKT Guiding Principles Partnership and Expertise [file 40900_2023_462_MOESM1_ESM.docx]

Supplementary File 1: IKT Guiding Principles Partnership and Expertise

| **Partner Members** | **Experience and Expertise** | | | | |
| --- | --- | --- | --- | --- | --- |
|  | **SCI Research System** | **Partnership Development** | **Evaluation Methods** | **KMb Science** | **End-of-Project KMb** |
| Anderson, K (academic; NASCIC) | ✓✓ | ✓✓ | ✓ | ✓✓ | ✓ |
| Athanasopolous, P (SCI ON) | ✓✓ | ✓✓ | ✓ | ✓ | ✓✓ |
| Campbell, M (PhD) |  | ✓ |  |  | ✓✓ |
| Chernesky, J (Praxis) | ✓✓ | ✓✓ |  |  | ✓✓ |
| Clarke, T | ✓✓ | ✓✓ | ✓ | ✓ | ✓✓ |
| Forwell, S | ✓✓ | ✓✓ | ✓ | ✓ | ✓✓ |
| Gainforth, H (academic; ICORD) | ✓✓ | ✓✓ | ✓✓ | ✓✓ | ✓✓ |
| Graham, I (academic) |  | ✓✓ | ✓✓ | ✓✓ | ✓✓ |
| Hoekstra, F (academic; ICORD) | ✓✓ | ✓✓ | ✓✓ | ✓✓ | ✓✓ |
| Kaiser, A (academic; CSRO) | ✓✓ | ✓✓ |  |  | ✓✓ |
| Maffin, J (SCI BC) | ✓✓ | ✓✓ | ✓ |  | ✓✓ |
| Martin Ginis, K (academic; ICORD) | ✓✓ | ✓✓ | ✓✓ | ✓✓ | ✓✓ |
| McBride, C (SCI BC) | ✓✓ | ✓✓ | ✓ | ✓ | ✓✓ |
| McPhail, L (ICORD) | ✓✓ | ✓✓ |  |  | ✓✓ |
| Mortenson, B (academic; ICORD) | ✓✓ | ✓✓ | ✓ |  | ✓ |
| Noonan, V | ✓✓ | ✓✓ | ✓ | ✓ | ✓ |
| Scarrow, G (MSHRBC) |  | ✓✓ | ✓ | ✓ | ✓✓ |
| Schaefer, L (academic) |  | ✓✓ | ✓ |  | ✓ |
| Sibley, K (academic) |  | ✓✓ | ✓✓ | ✓✓ | ✓✓ |
| Sweet, S (academic) | ✓✓ | ✓✓ | ✓✓ | ✓ | ✓✓ |
| West, C (academic; ICORD) | ✓✓ | ✓✓ |  |  | ✓✓ |
| Williams, R (GF Strong; ICORD) | ✓✓ | ✓✓ |  |  | ✓✓ |

Note. ✓ = experience; ✓✓ = expertise
