# Supplementary figures and images for: IKT Guiding Principles: demonstration of diffusion and dissemination in partnership
Source: Res Involv Engagem. 2023 Jul 12;9:53. doi: 10.1186/s40900-023-00462-1 (PMC10337125; doi:10.1186/s40900-023-00462-1)

Supplementary File 2: IKT Guiding Principles Partnership Governance Structure


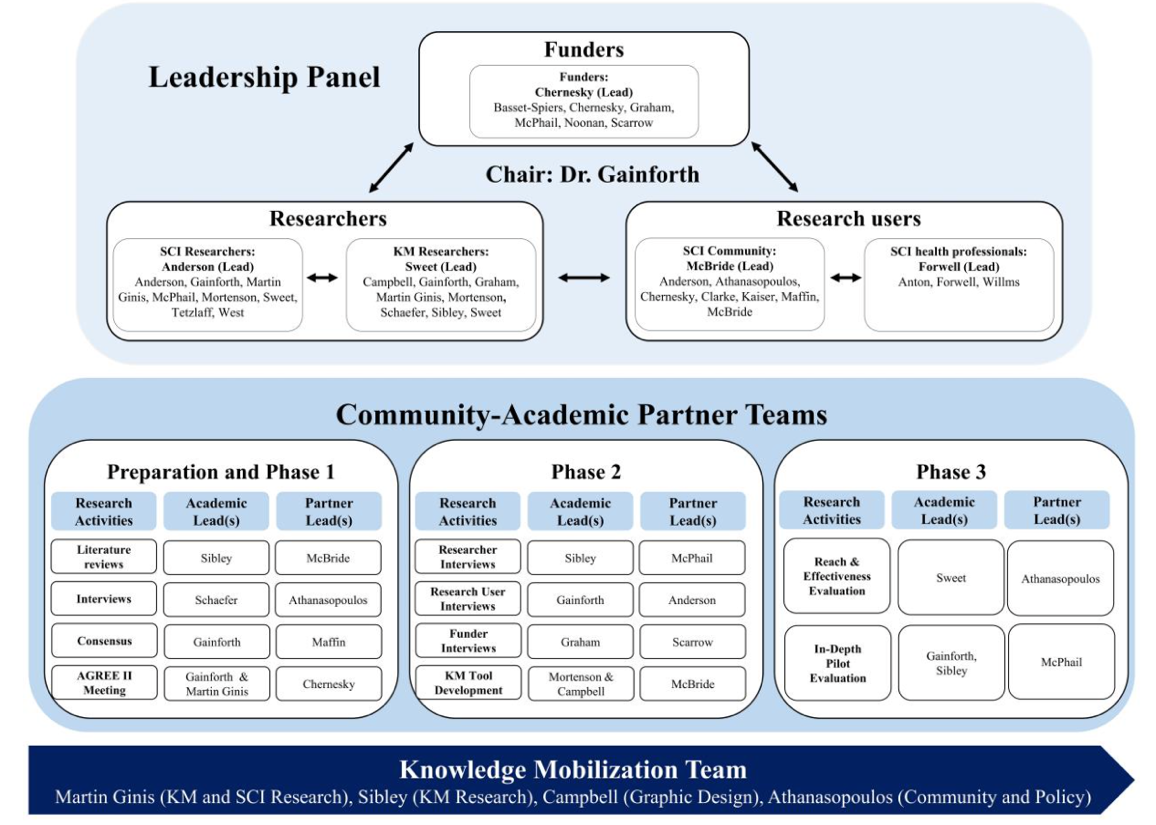

Supplement: Supplementary file 2 — Additional file 2. IKT Guiding Principles Partnership Governance Structure [file 40900_2023_462_MOESM2_ESM.docx]
