# Supplementary material for: IKT Guiding Principles: demonstration of diffusion and dissemination in partnership
Source: Res Involv Engagem. 2023 Jul 12;9:53. doi: 10.1186/s40900-023-00462-1 (PMC10337125; doi:10.1186/s40900-023-00462-1)
Supplement: Supplementary file 3 — Additional file 3. IKT Guiding Principles and Enacted Strategies [file 40900_2023_462_MOESM3_ESM.docx]

Supplementary File 3: IKT Guiding Principles and Enacted Strategies

| **IKT Strategies Used** | **Targeted IKT Guiding Principles*** |
| --- | --- |
| Flexible work hours and meeting times. | ***1 –*** Develop and maintain relationships.  ***7 –*** Address ethical considerations.  ***8 –*** Respect the practical considerations. |
| Flexibility for meeting availability by recording all discussions. | ***1 –*** Develop and maintain relationships.  ***7 –*** Address ethical considerations.  ***8 –*** Respect the practical considerations. |
| Partners were compensated for their time (in kind or cash). | ***4 –*** Value diverse expertise and knowledge.  ***6 –*** Meaningfully benefit.  ***7 –*** Address ethical considerations.  ***8 –*** Respect the practical considerations. |
| Partners engaged in identifying and prioritizing relevant dissemination goals, key messages, and processes. | ***2 –*** Share in decision-making.  ***4 –*** Value diverse expertise and knowledge.  ***5 –*** Flexible in tailoring research approach. |
| Partners engaged in dissemination activities (e.g., webinars, sending emails, posting on social media) to the extent they desired. | ***2 –*** Share in decision-making.  ***4 –*** Value diverse expertise and knowledge.  ***7 –*** Address ethical considerations.  ***8 –*** Respect the practical considerations. |
| Partners engaged in developing and authoring dissemination outputs. | ***2 –*** Share in decision-making.  ***4 –*** Value diverse expertise and knowledge.  ***6 –*** Meaningfully benefit.  ***7 –*** Address ethical considerations.  ***8 –*** Respect the practical considerations. |
| Partners engaged in establishing future dissemination priorities. | ***2 –*** Share in decision-making.  ***4 –*** Value diverse expertise and knowledge.  ***5 –*** Flexible in tailoring research approach. |
| Communication with partnership about dissemination occurred as frequently as requested by the partnership (i.e., bimonthly emails or when deadlines were near, quarterly meetings). | ***3 –*** Open communication.  ***4 –*** Value diverse expertise and knowledge. |
| Gathered information from partners about their thoughts on all processes, decisions, and next steps via different methods (e.g., interviews, surveys, group meetings). | ***2 –*** Share in decision-making.  ***5 –*** Flexible in tailoring research approach. |
| No decision about what to do next was made unless all partners could ‘live with’ the decision(s). Partners were asked for their opinion both over Zoom meetings and again over email to allow for personal reflection and change in opinion either way. | ***2 –*** Share in decision-making.  ***5 –*** Flexible in tailoring research approach. |
| Partners engaged in the partnership at the level they wanted (23). Flexibility around what each partner contributed (e.g., creating dissemination tools or just reviewing dissemination tools) was prioritized. | ***2 –*** Share in decision-making.  ***4 –*** Value diverse expertise and knowledge.  ***5 –*** Flexible in tailoring research approach.  ***6 –*** Meaningfully benefit. |
